# ESLI: Enhancing slope one recommendation through local information embedding

**DOI:** 10.1371/journal.pone.0222702

**Published:** 2019-10-10

**Authors:** Heng-Ru Zhang, Yuan-Yuan Ma, Xin-Chao Yu, Fan Min

**Affiliations:** School of Computer Science, Southwest Petroleum University, Chengdu, China; Universite du Quebec a Montreal, CANADA

## Abstract

Slope one is a popular recommendation algorithm due to its simplicity and high efficiency for sparse data. However, it often suffers from under-fitting since the global information of all relevant users/items are considered. In this paper, we propose a new scheme called enhanced slope one recommendation through local information embedding. First, we employ clustering algorithms to obtain the user clusters as well as item clusters to represent local information. Second, we predict ratings using the local information of users and items in the same cluster. The local information can detect strong localized associations shared within clusters. Third, we design different fusion approaches based on the local information embedding. In this way, both under-fitting and over-fitting problems are alleviated. Experiment results on the real datasets show that our approaches defeats slope one in terms of both mean absolute error and root mean square error.

## Introduction

Collaborative filtering (CF) [1–3] is one of the widely used techniques in recommender systems [4, 5]. CF does not rely on the content descriptions of items, but purely depends on preferences expressed by a set of users. Memory-based and model-based CF are two main approaches [3, 6]. The former uses the entire user-item database to make a prediction [7], such as slope one [8], *k*-nearest neighbor [9], and matrix factorization [10]. The latter first learns a descriptive model of user preferences and then uses it for predicting ratings [11], such as neural network classifiers [12], Bayesian network [13], linear classifiers [14].

Data sparsity [15] is one of the main factors affecting the prediction accuracy of CF. Slope one uses a linear regression model to handle data sparsity. By determining the quantitative relationship between two or more items, efficient recommendation can be generated in real time. However, slope one often faces under-fitting since the global information of all users/items are considered.

In this paper, we propose a new approach called enhanced slope one recommendation through local information embedding (ESLI). On one hand, we try to alleviate under-fitting caused by slope one with global information. This is fulfilled through using local information of users/items to accurately measure the similarities between two users’ preferences. On the other hand, we try to alleviate over-fitting caused by local information. This is fulfilled through appropriate granular selection [16] and approach fusion.

First, we employ clustering algorithms to extract local information. Users with similar rating habits will be clustered into one category. The user clusters represent local user information (LU). Correspondingly, items of similar popularity will be clustered into one category. The item clusters represent local item information (LI).

Second, we predict ratings using the local information of users and items in the same cluster. We design three enhanced slope-one approaches embedding local information. The local-user-global-item approach (LUGI, also called *A*_1_) only embeds user local information. The global-user-local-item approach (GULI, also called *A*_2_) only embeds item local information. The local-user-local-item approach (LULI, also called *A*_3_) embeds both the user and the item local information.

Third, we design four fusion approaches (*A*_4_, *A*_5_, *A*_6_, and *A*_7_) based on the above three basic approaches to make the best prediction. The four approaches merges LUGI, GULI, and LULI, respectively. We use the average of any two or three approaches to form four fusion approaches. In this way, both under-fitting and over-fitting are alleviated.

To examine the performance of the proposed method, we conducted experiments on the well-known MovieLens, DouBan datasets with a Java implementation. Experimental results show that (1) ESLI decreases both the mean absolute error (MAE) and root mean square error (RMSE) evaluation indicators; and (2) ELSI is more prominent than slope one in large datasets.

The rest of this paper is organized as follows: Firstly, we present the related works including rating system, slope one algorithm and clustering algorithms. Secondly, we discuss how to extract local information and embed it into the slope one algorithm. Subsequently, we present our experimental results for four datasets. Finally, we introduce the conclusion and further work. All code files and datasets are available from the Github database (https://github.com/FanSmale/ESLI.git) or Supporting Information (see S1 and S2 Files).

Table 1 defines notations used throughout the paper.

**Table 1:** Notations.

Notations	Meaning
*U*	The set of all users
*T*	The set of all items
*R*	The rating matrix
*m*	The number of users
*n*	The number of items
*r*_*i*,*j*_	The rating of *u*_*i*_ to *t*_*j*_
$\bar{r_{i,.}}$	The average rating of *u*_*i*_
$\bar{r_{.,j}}$	The average rating of *t*_*j*_
*g*	The *g*-th user cluster index
*q*	The *q*-th item cluster index
*G*_*g*_	The set of *g*-th user cluster
*Q*_*q*_	The set of *q*-th item cluster
*C*	The total number of user clusters
*E*	The total number of item clusters
*R*^*g*,.^	The sub-matrix ratings for *G*_*g*_ and *T*
*R*^.,*q*^	The sub-matrix ratings for *U* and *Q*_*q*_
*R*^*g*,*q*^	The sub-matrix ratings for *G*_*g*_ and *Q*_*q*_
$p_{i,j}^{g,.}$	The predicting rating of *u*_*i*_ to *t*_*j*_ corresponding to *g*-th user cluster
$p_{i,j}^{.,q}$	The predicting rating of *u*_*i*_ to *t*_*j*_ corresponding to *q*-th item cluster
$p_{i,j}^{g,q}$	The predicting rating of *u*_*i*_ to *t*_*j*_ corresponding to *g*-th user cluster and *q*-th item cluster
$f_{i,j}^{1}$	$(p_{i,j}^{g,.}+p_{i,j}^{.,q})/2$
$f_{i,j}^{2}$	$(p_{i,j}^{g,.}+p_{i,j}^{g,q})/2$
$f_{i,j}^{3}$	$(p_{i,j}^{.,q}+p_{i,j}^{g,q})/2$
$f_{i,j}^{4}$	$(p_{i,j}^{g,.}+p_{i,j}^{.,q}+p_{i,j}^{g,q})/3$

## Related work

The ESLI scheme uses the rating system and the local user/item information as input. The clustering algorithm is employed to obtain the local user/item information using the rating system.

### Rating system

Let *U* = {*u*_0_, *u*_1_, …, *u*_*m*−1_} be the set of users and *T* = {*t*_0_, *t*_1_, …, *t*_*n*−1_} be the set of items. The users’ ratings of the items form a rating matrix. The rating function is given by [17]

$$\begin{array}{c} R:U\times T\to V, \end{array}$$ (1)

where *V* is the rating scale. For convenience, we denote the rating system as an *m* × *n* rating matrix *R* = (*r*_*i*,*j*_)_*m*×*n*_, where *r*_*i*,*j*_ = *R*(*u*_*i*_, *t*_*j*_), 0 ≤ *i* ≤ *m* − 1, and 0 ≤ *j* ≤ *n* − 1.

Table 2 depicts an example of rating system, where *m* = 5, *n* = 5 and *V* = {1, 2, …, 10}. “–” indicates that the users do not have ratings on the items.

**Table 2:** The rating matrix (*R*).

UID/TID	*t*_0_	*t*_1_	*t*_2_	*t*_3_	*t*_4_
*u*_0_	2	4	–	5	1
*u*_1_	7	9	8	6	9
*u*_2_	–	8	9	8	6
*u*_3_	6	–	9	8	7
*u*_4_	1	3	5	4	2

### Slope one

The underlying principle of the slope one algorithm [8] is based on linear regression to determine the extent by which users prefer one item to another. It uses a simple formula *f*(*x*) = *x* + *b*, where the parameter *b* represents the average deviation of the ratings of two users or items [8]. Then, given a user’s ratings of certain items, we can predict the user’s ratings of other items based on the average deviation.

Slope one [8] is adaptive to data sparsity. It is easy to realize and extend. Due to it can generate effective recommendation in real time, it is used in many online recommender systems, such as movies, music and books. However, owing to calculate the average deviation with global information, this can lead to under-fitting problem.

### Global and local rating information fusion

CF uses rating information to predict users’ preferences for items [18–21]. Rating information can be collected by implicit means, explicit means or both. Implicit ratings are inferred from a user’s behaviors. In the explicit collection of ratings, the user is asked to provide an opinion about the item on a rating scale. Explicit ratings provide a more accurate description of a user’s preference for an item than implicit ratings. We only take explicit ratings as input in this paper.

CF algorithms typically use global or local information about user preferences to help people make choices. Some studies take global information as input, such as slope one [8], matrix factorization [22], which leads to under-fitting problems. Some studies take local information as input, such as MG-LCR [23], UPUC-CF [24], which leads to over-fitting problems. In order to avoid the above two problems, some studies combine local and global information to learn models, such as MPMA [25], GLOMA [26]. However, to the best of our knowledge, the fusion of global and local information has not been used for slope one algorithm.

### Clustering algorithms

Clustering is used to reveal the intrinsic properties and laws of data [27, 28]. It attempts to divide the data sample into several subsets which are usually not intersected [28]. In collaborative filtering, users and items can be grouped into different clusters. User-based clustering [29] divides users with similar rating habits into a cluster. Item-based clustering [3] divides items into different clusters based on the similarity of attributes such as item popularity, etc.

There are a lot of clustering algorithms, such as *k*-means [30] and M-distance [31]. *k*-means [30] randomly selects *k* samples as the center points and obtains clusters through multiple iterations. It is easy to implement, but the convergent speed is slow and the clustering results are uncertain. M-distance [31] defines the relationship between users or items using the average rating. Compared with *k*-means clustering, its convergent speed is fast and the clustering results are deterministic.

## ESLI scheme

In this section, we describe our proposed scheme. Firstly, we describe the extraction of local information. Then, we describe the ESLI scheme, which includes three basic approaches and four fusion approaches.

### Local information extraction

Local information is intended to extract the rating habits of similar users or the popularity of similar items. Naturally, the clustering algorithm is employed to obtain it. LU/LI are used to represent the local user/item information, respectively. S1 Fig depicts the schematic diagram of local information extraction.

S1A Fig depicts an example of LU. Users are classified into different clusters based on the rating habits. The first user cluster is composed of *u*_0_ and *u*_4_. Their ratings are no more than 5 points for all items. They are more strict users and are used to providing the lower rating. The second user cluster is composed of *u*_1_, *u*_2_ and *u*_3_. Their ratings are no less than 6 points for all items. They are more tolerant users and are used to providing the higher rating.

S1B Fig depicts an example of LI. Items are classified into different clusters based on the items popularity. The first item cluster is composed of *t*_0_ and *t*_4_. They get a lot of low ratings of 1-2 points. The low ratings indicate that they are less popular. The second item cluster is composed of *t*_1_, *t*_2_ and *t*_3_. They get a lot of high ratings of 8-9 points. The high ratings indicate that they are popular items.

S1C Fig depicts an example of LULI. Each cluster contains a subset of users and a subset of items. The first cluster is composed of a user group {*u*_0_, *u*_4_} and an item group {*t*_0_, *t*_4_}. They are the lowest rating of 1-2 points. The second cluster is composed of a user group {*u*_0_, *u*_4_} and an item group {*t*_1_, *t*_2_, *t*_3_}. They are the lower rating of 3-5 points. The third cluster is composed of a user group {*u*_1_, *u*_2_, *u*_3_} and an item group {*t*_0_, *t*_4_}. They are the higher rating of 6-7 points. The fourth cluster is composed of a user group {*u*_1_, *u*_2_, *u*_3_} and an item group {*t*_1_, *t*_2_, *t*_3_}. They are the highest rating of 8-9 points. Within each LULI cluster, the rating distribution is more balanced and the rating similarity is higher than LUGI and GULI.

### Enhanced slope one algorithms

S2 Fig lists eight slope one approaches. S2A Fig depicts global-user-global-item approach (GUGI) [8]. S2B–S2D Fig depict three basic approaches, including LUGI, GULI and LULI. S2E–S2H Fig depict four fusion approaches.

Approach *A*_1_ uses the sub-matrix *R*^*g*,.^ as input, and computes the predicted rating $p_{i,j}^{g,.}$ for *u*_*i*_ to *t*_*j*_ as

$$\begin{array}{cc} p_{i,j}^{g,.} & =\frac{\sum_{j^{\prime }=0}^{m-1}\frac{\sum_{u_{i^{\prime }}\in G_{g}}\left(r_{i^{\prime },j}-r_{i^{\prime },j^{\prime }}\right)}{\left|\{u_{i^{\prime }}\in G_{g}|r_{i^{\prime },j}>0,r_{i^{\prime },j^{\prime }}>0\}\right|}}{\left|\{t_{j^{\prime }}|r_{i,j^{\prime }}>0\}\right|}. \end{array}$$ (2)

Based on S2B Fig, we have

**Example 1** $p_{3,1}^{1,.}=\frac{\left(9-7+6)+(\frac{\left(8-9)+(9-8\right)}{2}+9)+(\frac{\left(8-8)+(9-9\right)}{2}+8)+(\frac{\left(8-6)+(9-6\right)}{2}+7\right)}{4}\approx 8.6$.

Approach *A*_2_ uses sub-matrix *R*^.,*q*^ as input, and computes the predicted rating $p_{i,j}^{.,q}$ for *u*_*i*_ to *t*_*j*_ as

$$\begin{array}{cc} p_{i,j}^{.,q} & =\frac{\sum_{j^{\prime }\in Q_{q}}\frac{\sum_{i^{\prime }=0}^{n-1}\left(r_{i^{\prime },j}-r_{i^{\prime },j^{\prime }}\right)}{\left|\{u_{i^{\prime }}|r_{i^{\prime },j}>0,r_{i^{\prime },j^{\prime }}>0\}\right|}}{\left|\{t_{j^{\prime }}|t_{j^{\prime }}\in Q_{q},r_{i,j^{\prime }}>0\}\right|}. \end{array}$$ (3)

Based on S2C Fig, we have

**Example 2** $p_{3,1}^{.,1}=\frac{\left(\frac{\left(4-5)+(9-9)+(8-8)+(3-4\right)}{4}+8)+(\frac{\left(9-8)+(8-9)+(3-5\right)}{3}+9\right)}{2}\approx 7.9$.

Approach *A*_3_ uses the sub-matrix *R*^*g*,*q*^ as input, and computes the predicted rating $p_{i,j}^{g,q}$ for *u*_*i*_ to *t*_*j*_ as

$$\begin{array}{c} p_{i,j}^{g,q}=\frac{\sum_{j^{\prime }\in Q_{q}}\frac{\sum_{i^{\prime }\in G_{g}}\left(r_{i^{\prime },j}-r_{i^{\prime },j^{\prime }}\right)}{\left|\{u_{i^{\prime }}|r_{i^{\prime },j}>0,r_{i^{\prime },j^{\prime }}>0,i^{\prime }\in G_{g}\}\right|}}{\left|\{t_{j^{\prime }}|r_{i^{\prime },j^{\prime }}>0,j^{\prime }\in Q_{q},i^{\prime }\in G_{g}\}\right|}. \end{array}$$ (4)

Based on S2D Fig, we have

**Example 3** $p_{3,1}^{1,1}=\frac{\left(\frac{\left(8-8)+(9-9\right)}{2}+8)+(\frac{\left(8-9)+(9-8\right)}{2}+9\right)}{2}=8.5$.

The algorithm *A*_4_ takes the average of the approaches *A*_1_ and *A*_2_ as the final predicted rating

$$\begin{array}{c} f_{i,j}^{1}=\frac{p_{i,j}^{g,.}+p_{i,j}^{,.q}}{2}. \end{array}$$ (5)

Based on Example 1 and 2, we have

**Example 4** $f_{3,1}^{1}=\frac{p_{3,1}^{1,.}+p_{3,1}^{.,1}}{2}=\frac{8.6+7.9}{2}\approx 8.3$.

The algorithm *A*_5_ takes the average of the approaches *A*_1_ and *A*_3_ as the final predicted rating

$$\begin{array}{c} f_{i,j}^{2}=\frac{p_{i,j}^{g,.}+p_{i,j}^{g,q}}{2}. \end{array}$$ (6)

Based on Example 1 and 3, we have

**Example 5** $f_{3,1}^{2}=\frac{p_{3,1}^{1,.}+p_{3,1}^{1,1}}{2}=\frac{8.6+8.5}{2}\approx 8.6$.

The algorithm *A*_6_ takes the average of the approaches *A*_2_ and *A*_3_ as the final prediction rating

$$\begin{array}{c} f_{i,j}^{3}=\frac{p_{i,j}^{.,q}+p_{i,j}^{g,q}}{2}. \end{array}$$ (7)

Based on Example 2 and 3, we have

**Example 6** $f_{3,1}^{3}=\frac{p_{3,1}^{.,1}+p_{3,1}^{1,1}}{2}=\frac{7.9+8.5}{2}=8.2$.

The approach *A*_7_ takes the average of the approaches *A*_1_, *A*_2_ and *A*_3_ as the final prediction rating

$$\begin{array}{c} f_{i,j}^{4}=\frac{p_{i,j}^{g,.}+p_{i,j}^{.,q}+p_{i,j}^{g,q}}{3}. \end{array}$$ (8)

Based on Example 1, 2 and 3, we have

**Example 7** $f_{i,j}^{4}=\frac{p_{3,1}^{1,.}+p_{3,1}^{.,1}+p_{3,1}^{1,1}}{3}=\frac{8.6+7.9+8.5}{3}\approx 8.3$.

### Time complexity analysis

Let the number of users and items be *m* and *n*, respectively. The complexity analysis includes off-line and on-line phases. Local information can be extracted in the off-line phase by clustering algorithm. For M-distance clustering algorithm [31], the time complexity is *O*(*mn*).

In the on-line prediction stage, we discuss the time complexity of predicting a rating. For global-user-global-item approach (GUGI), the time complexity is *O*(*mn*). Let the number of user groups and item groups be *C* and *E*, respectively. The time complexity of local-user-global-item approach (LUGI) is $O(\frac{mn}{C})$. The time complexity of global-user-local-item approach (GULI) is $O(\frac{mn}{E})$. The time complexity of local-user-local-item approach (LULI) is $O(\frac{mn}{CE})$.

## Experiments

In this section, we report extensive computational tests designed to address the following questions:

1. Does the ESLI model perform better than existing slope one [8] in terms of MAE and RMSE?
2. Does the ESLI model have a more prominent advantage than the existing slope one [8] when there are more users or items?

Question 1 compares the MAE and RMSE between our proposed scheme and existing slope one. The question is the core issue of this paper.

Question 2 compares the MAE and RMSE between our proposed scheme and the existing slope one under different scale of users or items.

### Datasets

Table 3 lists the basic information of Movielens 100K (ML100K), Movielens 1M (ML1M), Movielens 10M (ML10M) and DouBan [32] (DB, https://www.cse.cuhk.edu.hk/irwin.king.new/pub/data/douban) datasets. The number of users ranges from 943 to 71,567. The number of items ranges from 1,682 to 39,695. The number of ratings ranges from 100,000 to 10,000,054, while the density of rating ranges from 0.78% to 6.30%. The average rating ranges from 3.51 to 3.75.

**Table 3:** The basic information of four datasets.

Dataset	# of Users	# of Items	# of Ratings	# of Density	# of Average rating
ML100K	943	1,682	100,000	6.30%	3.53
ML1M	6,040	3,900	1,000,209	4.19%	3.59
ML10M	71,567	10,681	10,000,054	1.31%	3.51
DB	2,965	39,695	912,479	0.78%	3.75

The rating distributions of four datasets have similar normal distribution characteristics. The rating scale ranges from 0.5 to 5. The highest scale is 5. The lowest scale is 0.5. The step length is 0.5. The frequency is the highest when the rating is 4, and the frequency is the second highest when the rating is 3 or 5. For the ML100K dataset, the maximum number of ratings for users/movies are 737/583, respectively, with a minimum of 168/1, respectively. For the ML1M dataset, the maximum number of ratings for users/movies are 2,314/3,428, respectively, with a minimum of 341/388, respectively. For the ML10M dataset, the maximum number of ratings for users/movies are 7,359/34,864, respectively, with a minimum of 20/1, respectively. For the DB dataset, the maximum number of ratings for users/movies are 10,157/1,274, respectively, with a minimum of 166/1, respectively.

### Evaluation metrics

We employ MAE [33, 34] and RMSE [34, 35] as evaluation metrics. The lower the values of MAE and RMSE, the better the performance of the recommender system [36].

Given a rating system, the MAE is calculated by

$$\begin{array}{c} \text{MAE}=\frac{\sum_{0}^{m-1}\sum_{0}^{n-1}\left|r_{i,j}-p_{i,j}\right|}{\left|\{\left\langle i,j\right\rangle |0\leq i<m,0\leq j<n,r_{i,j}>0\}\right|}, \end{array}$$ (9)

and the RMSE is computed by

$$\begin{array}{c} \text{RMSE}=\sqrt{\frac{\sum_{0}^{m-1}\sum_{0}^{n-1}{\left(r_{i,j}-p_{i,j}\right)}^{2}}{\left|\{\left\langle i,j\right\rangle |0\leq i<m,0\leq j<n,r_{i,j}>0\}\right|}}, \end{array}$$ (10)

where *p*_*i*,*j*_ is the prediction rating of *u*_*i*_ for *t*_*j*_.

### Experimental design

We design two sets of experiments to answer the questions raised at the beginning of this section.

*Exp1*. We first determine the parameters *C* and *E*, and then obtain the optimal MAE and RMSE. We employ *k*-means and M-distance clustering algorithms to extract user and item local information. To determine the parameters, we change *C* ∈ [2, 10] and *E* ∈ [2, 10] and obtain the minimum MAE and RMSE.

*Exp2*. Our aim is to analyze its impact on the ESLI scheme under different scale of users and items. First, we gradually increase the number of users under the condition that all items are involved. Second, we gradually increase the number of items under the condition that all users are involved.

We randomly divide the entire dataset into a training set and a testing set. 80% of the data are usually specified as a training set and the remaining 20% as a testing set.

### Sensitivity to parameters

Granular selection is one of the important factors affecting the performance of the ESLI scheme [37–39].

Because the approach *A*_7_ is a fusion algorithm for all basic approaches. We find the optimal *C* and *E* through computing the MAE of approach *A*_7_. S3 and S4 Figs show the MAE of approach *A*_7_ when *C* ∈ [2, 10] and *E* ∈ [2, 10] for the ML1M dataset.

In S3 Fig, the number of item clusters is set to 3. When the user cluster *C* ∈ [2, 4], the MAE decreases. When the user cluster *C* ∈ [4, 10], the MAE increases. We get the minimum MAE when *C* = 4. In S4 Fig, the number of user clusters is set to 4. When the user cluster *E* ∈ [2, 3], *E* ∈ [4, 5] and *E* ∈ [8, 10], the MAE decreases. When the user cluster *E* ∈ [3, 4] and *E* ∈ [5, 8], the MAE increases. We get the minimum MAE when *E* = 3.

We analyze the performance of the ESLI through changing the number of users/items. S5 and S6 Figs show the MAE comparison between *A*_7_ and GUGI. In S5 Fig, we fix the number of items, then gradually increase the number of users. As the number of users increases, the advantages of the ESLI scheme become more apparent. In S6 Fig, we fix the number of users, then gradually increase the number of items. As the number of items increases, the advantages of the ESLI scheme become more apparent.

### Runtime comparison

The time complexities of GUGI, LUGI (*A*_1_), GULI (*A*_2_), and LULI (*A*_3_) is *O*(*mn*), $O(\frac{mn}{C})$, $O(\frac{mn}{E})$, and $O(\frac{mn}{CE})$, respectively. Therefore we expect the runtime of LUGI, GULI, and LULI is 1/*C*, 1/*E*, and 1/*CE* of GUGI. When *C* = 4 and *E* = 3, they should be 1/4, 1/3, and 1/12, respectively.

The runtime of all algorithms under M-distance clustering is compared in Table 4. Note that the runtime is the total execution time, which includes the file input and output overhead. The computations were performed on a Windows 10 64-bit operating system with 8 GB RAM and intel Core i5 CPU@3.4GHz processors, using java software.

**Table 4:** Runtime comparison under M-distance clustering (unit: ms).

Algorithms	Dataset			
	ML100K	ML1M	ML10M	DB
*GUGI*	6,295	92,499	9,278,753	78,398
*A*_1_	4,061	53,109	5,334,255	53,159
*A*_2_	4,926	55,990	5,468,273	55,923
*A*_3_	3,617	39,643	4,073,868	43,139
*A*_4_	7,638	98,433	9,075,662	85,890
*A*_5_	9,945	106,902	11,450,463	90,936
*A*_6_	9,839	106,844	11,336,946	92,349
*A*_7_	19,777	255,122	20,658,793	165,813

For ML100K dataset, the experimental values are 41/63, 49/63, and 36/63, respectively. For ML1M dataset, the experimental values are 53/92, 56/92, and 40/92, respectively. For ML10M dataset, the experimental values are 53/93, 55/93, and 41/93, respectively. For DB dataset, the experimental values are 53/78, 56/78, and 43/78, respectively. They generally comply with the expected values. *A*_4_, *A*_5_, *A*_6_ and *A*_7_ are fusion algorithms, therefore they have more runtime than GUGI.

### Comparison of MAE and RMSE

We compare the performance between ESLI scheme and the traditional slope one in terms of MAE and RMSE.

Table 5 shows MAE comparison under M-distance clustering.

**Table 5:** MAE comparison under M-distance clustering.

Algorithms	Dataset			
	ML100K	ML1M	ML10M	DB
*GUGI*	0.7417±0.0047	0.7713±0.0023	0.7624±0.0052	0.6632±0.0045
*A*_1_	0.7594±0.0029	0.7474±0.0033	**0.7273**±**0.0008**	0.6792±0.0064
*A*_2_	**0.7401**±**0.0043**	0.7751±0.0020	0.7641±0.0004	0.6634±0.0045
*A*_3_	0.7575±0.0029	0.7489±0.0035	0.7301±0.0050	**0.6522**±**0.0057**
*A*_4_	0.7552±0.0027	**0.7473**±**0.0032**	0.7276±0.0009	0.6716±0.0061
*A*_5_	0.7470±0.0031	0.7516±0.0030	0.7349±0.0007	0.6659±0.0050
*A*_6_	0.7452±0.0027	0.7532±0.0028	0.7361±0.0007	0.6585±0.0045
*A*_7_	0.7433±0.0027	0.7520±0.0029	0.7351±0.0008	0.6660±0.0050
Lower	0.21%	3.11%	4.60%	1.66%

For dataset ML100K, approach *A*_2_ obtains the lowest MAE, which is 0.21% lower than the traditional GUGI approach. For dataset ML1M, approach *A*_4_ obtains the lowest MAE, which is 3.11% lower than the traditional GUGI approach. For dataset ML10M, approach *A*_1_ obtains the lowest MAE, which is 4.60% lower than the traditional GUGI approach. For dataset DB, approach *A*_3_ obtains the lowest MAE, which is 1.66% lower than the traditional GUGI approach.

Table 6 shows RMSE comparison under M-distance clustering.

**Table 6:** RMSE comparison under M-distance clustering.

Algorithms	Dataset			
	ML100K	ML1M	ML10M	DB
*GUGI*	**0.9424**±**0.0066**	0.9670±0.0034	0.9729±0.0008	0.8633±0.0067
*A*_1_	0.9804±0.0079	**0.9423**±**0.0045**	**0.9317**±**0.0008**	0.9134±0.0098
*A*_2_	0.9439±0.0073	0.9714±0.0031	0.9750±0.0007	0.8631±0.0063
*A*_3_	0.9802±0.0090	0.9430±0.0043	0.9354±0.0067	0.8586±0.0083
*A*_4_	0.9754±0.0079	0.9419±0.0044	0.9319±0.0008	0.8931±0.0089
*A*_5_	0.9570±0.0069	0.9441±0.0043	0.9397±0.0008	0.8758±0.0076
*A*_6_	0.9568±0.0074	0.9476±0.0051	0.9409±0.0007	**0.8547**±**0.0062**
*A*_7_	0.9531±0.0068	0.9442±0.0043	0.9401±0.0007	0.8758±0.0076
Lower	-0.16%	2.55%	4.23%	1.00%

For dataset ML100K, all ESLI approaches are no lower than the traditional GUGI approach. For dataset ML1M, approach *A*_1_ obtains the lowest RMSE, which is 2.55% lower than the traditional GUGI approach. For dataset ML10M, approach *A*_1_ obtains the lowest RMSE, which is 4.23% lower than the traditional GUGI approach. For dataset DB, approach *A*_6_ obtains the lowest RMSE, which is 1.00% lower than the traditional GUGI approach.

Table 7 shows MAE comparison under *k*-means clustering.

**Table 7:** MAE comparison under *k*-means clustering.

Algorithms	Dataset			
	ML100K	ML1M	ML10M	DB
*GUGI*	**0.7417**±**0.0047**	0.7713±0.0023	0.7624±0.0052	**0.6632**±**0.0045**
*A*_1_	0.7694±0.0059	0.7709±0.0016	0.7614±0.0013	0.6708±0.0067
*A*_2_	0.7431±0.0048	0.7766±0.0020	**0.7574**±**0.0009**	0.6980±0.0086
*A*_3_	0.7717±0.0059	0.7755±0.0011	0.7652±0.0049	0.6634±0.0040
*A*_4_	0.7661±0.0056	0.7726±0.0013	0.7585±0.0008	0.6904±0.0080
*A*_5_	0.7517±0.0060	**0.7697**±**0.0017**	0.7597±0.0010	0.6822±0.0068
*A*_6_	0.7510±0.0020	0.7747±0.0013	0.7624±0.0011	0.6748±0.0062
*A*_7_	0.7485±0.0057	0.7716±0.0015	0.7609±0.0009	0.6823±0.0068
Lower	-0.19%	0.21%	0.66%	-0.03%

For datasets ML100K and DB, all ESLI approaches are no lower than the traditional GUGI approach. For dataset ML1M, approach *A*_5_ obtains the lowest MAE, which is 0.21% lower than the traditional GUGI approach. For dataset ML10M, approach *A*_2_ obtains the lowest MAE, which is 0.66% lower than the traditional GUGI approach.

Table 8 shows RMSE comparison under *k*-means clustering.

**Table 8:** RMSE comparison under *k*-means clustering.

Algorithms	Dataset			
	ML100K	ML1M	ML10M	DB
*GUGI*	**0.9424**±**0.0066**	0.9670±0.0034	0.9729±0.0008	**0.8633**±**0.0067**
*A*_1_	0.9887±0.0079	0.9711±0.0007	**0.9693**±**0.0012**	0.9543±0.0181
*A*_2_	0.9469±0.0076	0.9733±0.0030	0.9764±0.0008	0.8635±0.0066
*A*_3_	0.9941±0.0088	0.9762±0.0010	0.9745±0.0045	0.9015±0.0132
*A*_4_	0.9844±0.0074	0.9728±0.0008	0.9707±0.0010	0.9347±0.0164
*A*_5_	0.9589±0.0078	**0.9667**±**0.0014**	0.9703±0.0010	0.9041±0.0129
*A*_6_	0.9626±0.0085	0.9724±0.0009	0.9736±0.0008	0.8836±0.0110
*A*_7_	0.9553±0.0073	0.9688±0.0011	0.9718±0.0008	0.9042±0.0128
Lower	-0.49%	0.03%	0.37%	-0.02%

For datasets ML100K and DB, all ESLI approaches are no lower than the traditional GUGI approach. For dataset ML1M, approach *A*_5_ obtains the lowest RMSE, which is 0.03% lower than the traditional GUGI approach. For dataset ML10M, approach *A*_1_ obtains the lowest RMSE, which is 0.37% lower than the traditional GUGI approach.

In general, the M-distance-based ESLI is superior to the *k*-means-based ESLI. The *k*-means clustering is non-deterministic and is related to the initial center and distance function. The M-distance clustering is deterministic and is only relevant to the average rating of the user/item. The user average rating indicates her/his rating preference, and the item average score indicates its popularity. Compared with the *k*-means clustering method, the M-distance clustering method can better reflect the difference in ratings between different clusters.

## Conclusion and further work

In this paper, we propose an ESLI scheme for local information extraction based on clustering. In the ESLI scheme, we design seven different local information embedding approaches. The experimental results show that our scheme is better than slope one in terms of both MAE and RMSE.

In the future, we will apply the concept of local information embedding to other collaborative filtering algorithms. For model-based recommendation algorithms, the local demographic and occupation information will be considered.

## Supporting information

S1 File ML100K. (ZIP)

S2 File StableMA-master. (JAR)

S1 Fig (TIF)

S2 Fig (TIF)

S3 Fig (TIF)

S4 Fig (TIF)

S5 Fig (TIF)

S6 Fig (TIF)
