# Supplementary figures and images for: ESLI: Enhancing slope one recommendation through local information embedding
Source: PLoS One. 2019 Oct 10;14(10):e0222702. doi: 10.1371/journal.pone.0222702 (PMC6786606; doi:10.1371/journal.pone.0222702)

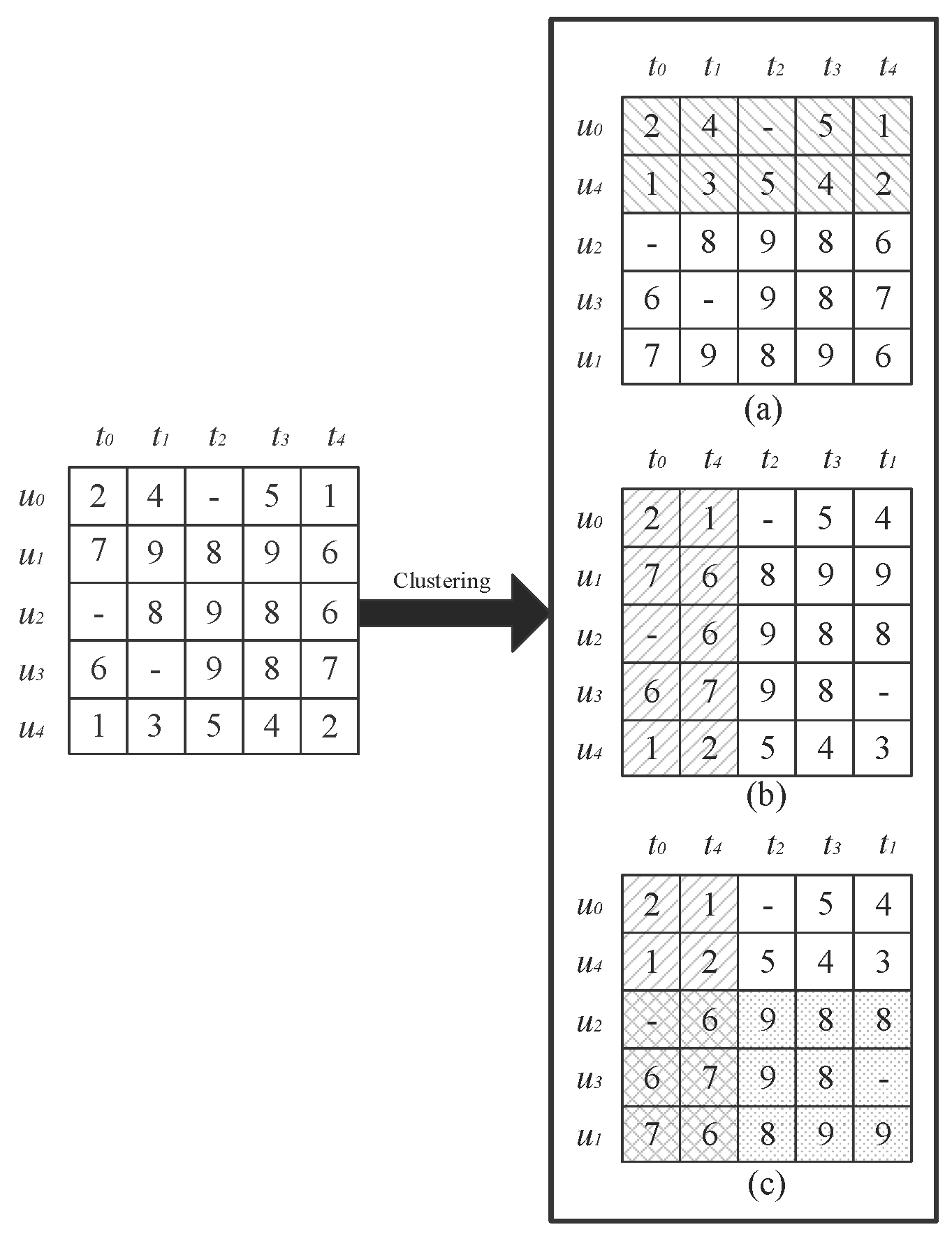

Supplement: S1 Fig — (TIF) [file pone.0222702.s003.tif]

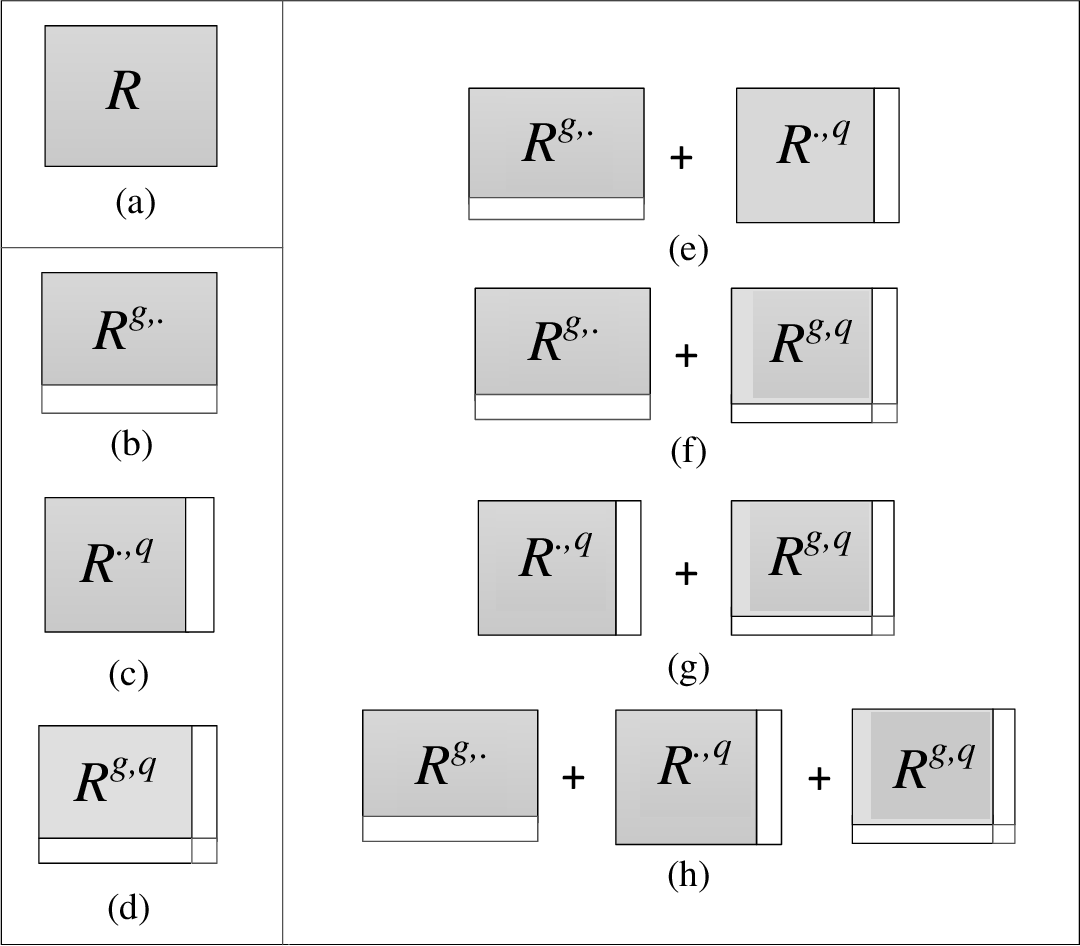

Supplement: S2 Fig — (TIF) [file pone.0222702.s004.tif]

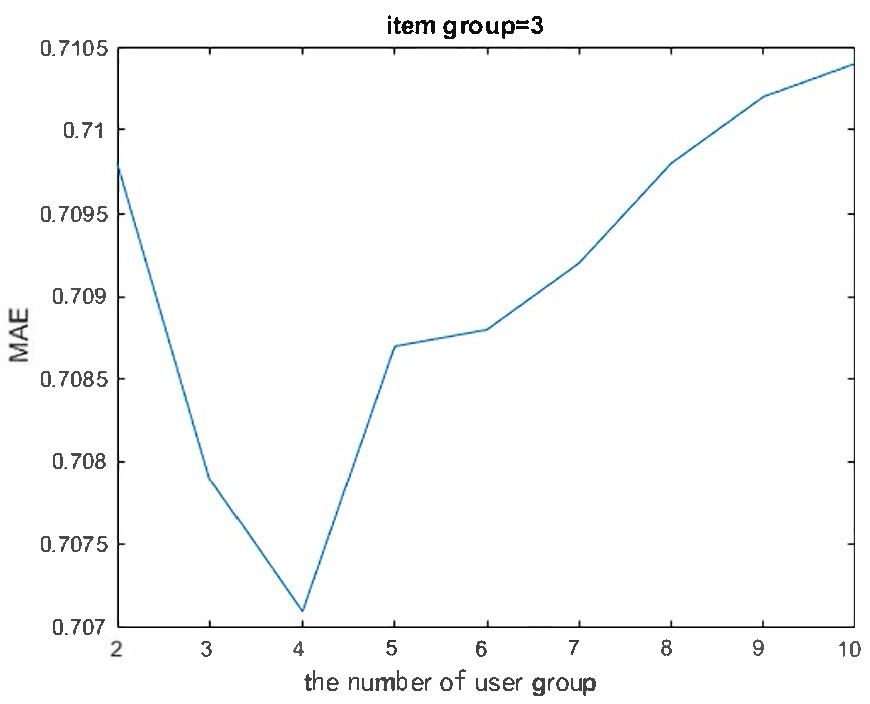

Supplement: S3 Fig — (TIF) [file pone.0222702.s005.tif]

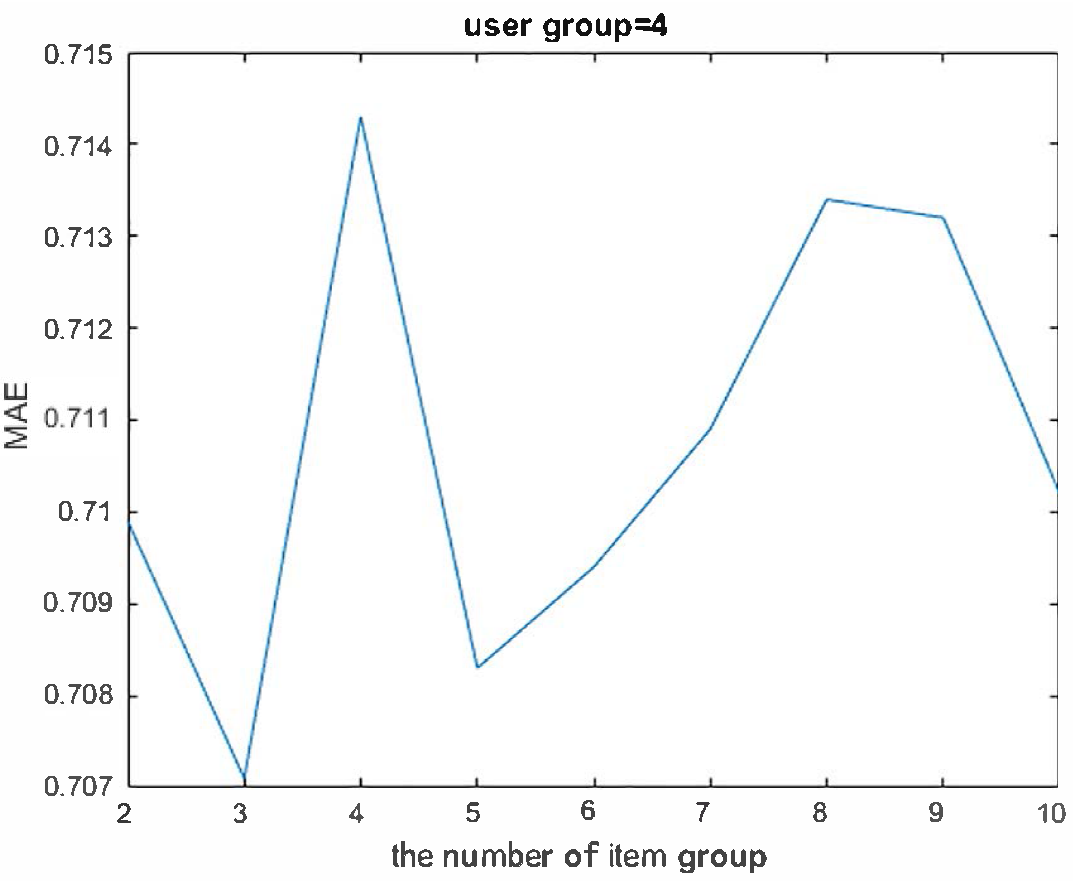

Supplement: S4 Fig — (TIF) [file pone.0222702.s006.tif]

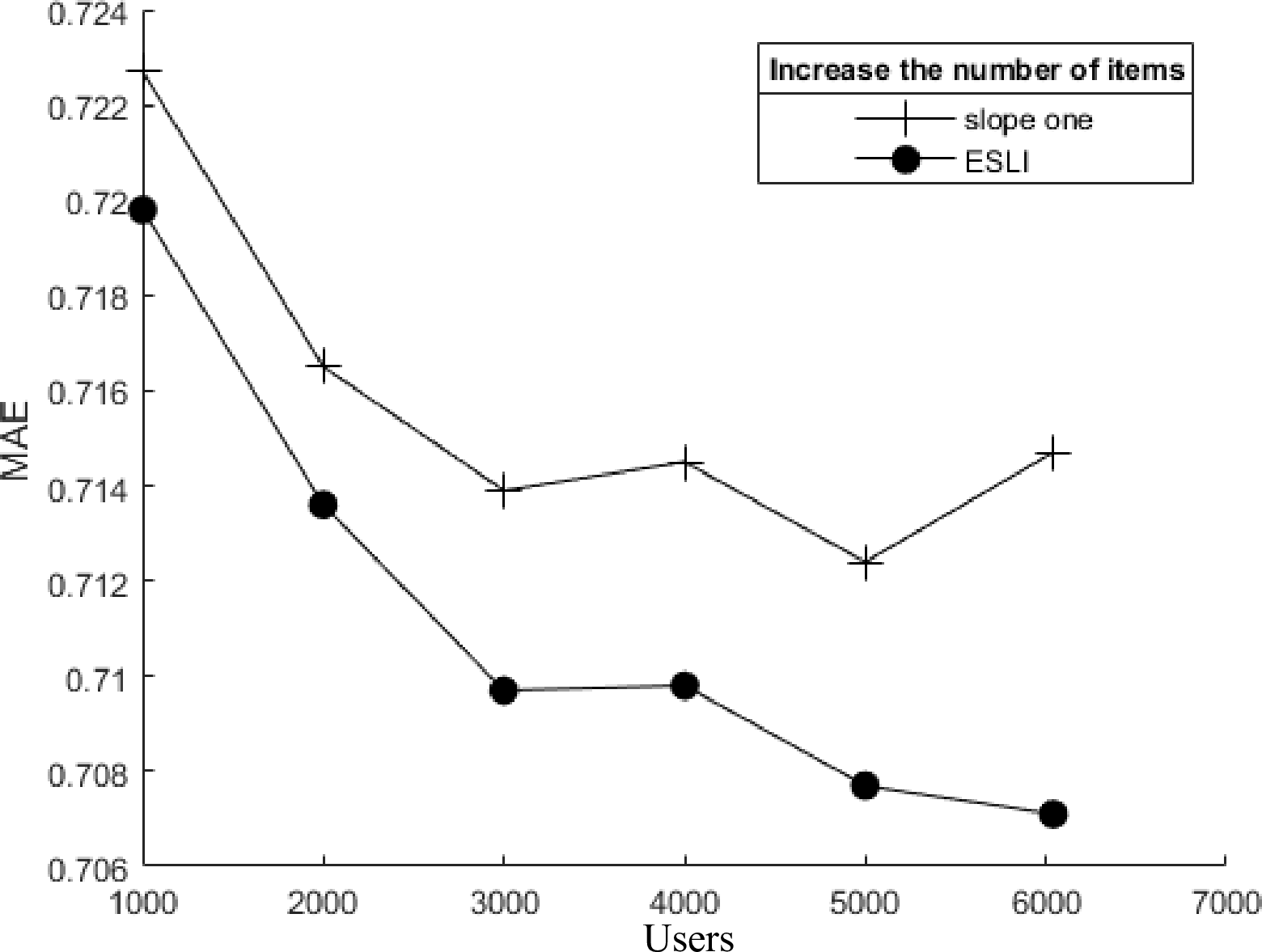

Supplement: S5 Fig — (TIF) [file pone.0222702.s007.tif]

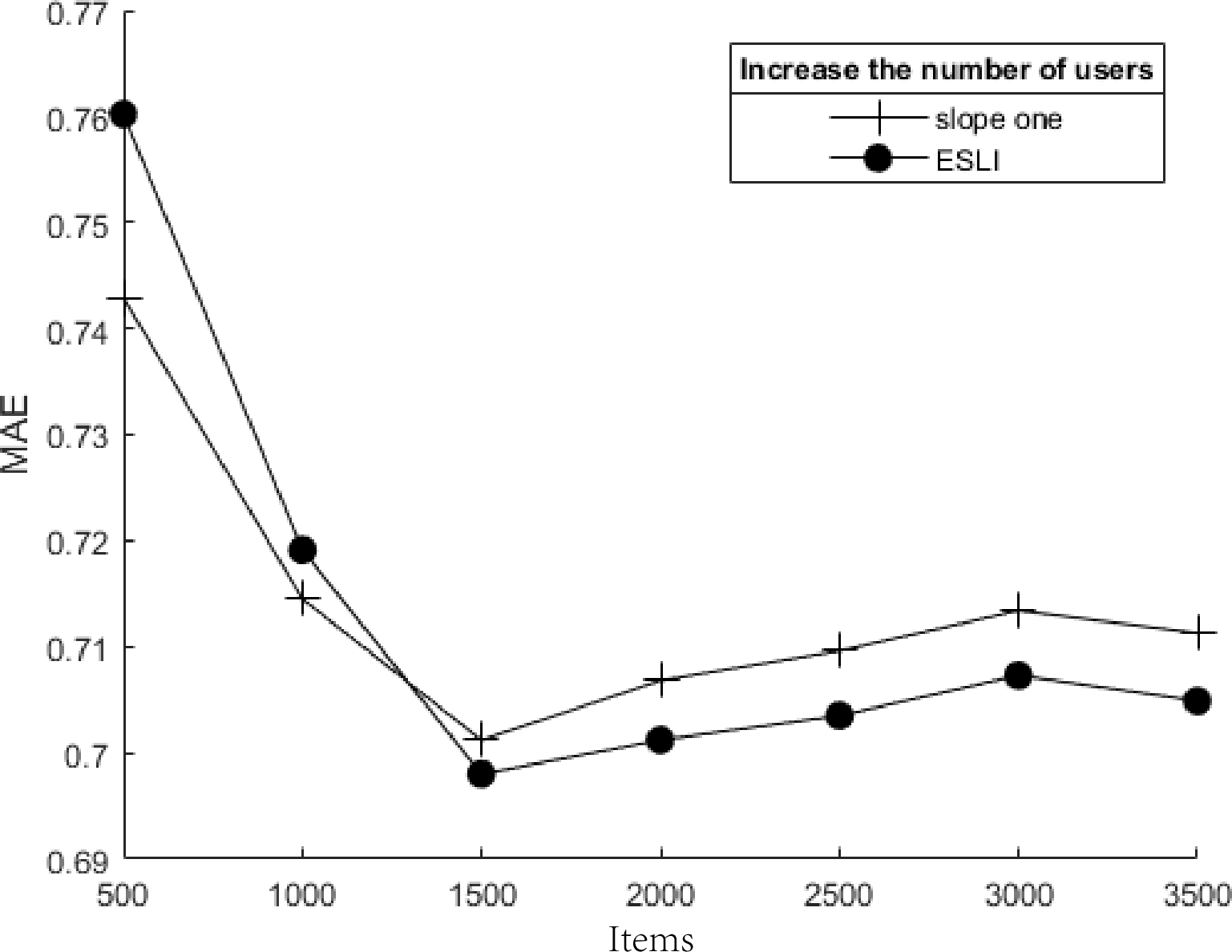

Supplement: S6 Fig — (TIF) [file pone.0222702.s008.tif]
